# Computational modelling of graphene/aluminum nitride (GP/AlN) hybrid materials for the detection of 2,4 dichlorophenoxyacetic acid (DCP) pollutant

**DOI:** 10.1039/d4ra03345k

**Published:** 2024-07-10

**Authors:** Chioma G. Apebende, Ismail O. Amodu, Miracle N. Ogbogu, Ubua P. Unimuyi, Monsurat Alarape Raimi, Godwin O. Igomah

**Affiliations:** a Department of Pure and Applied Chemistry, University of Calabar Calabar Nigeria gloriaapebende@gmail.com; b Department of Mathematics and Statistics, University of Calabar Calabar Nigeria; c Department of Genetics and Biotechnology, University of Calabar Calabar Nigeria; d Department of Chemistry, University of Ilorin Ilorin Nigeria; e Department of Physics, University of Calabar Calabar Nigeria

## Abstract

Despite their efficacy in eliminating undesired crops and increasing yield, a range of environmental issues and chronic ailments arise when hazardous chemicals are highly concentrated in wastewater and then deposited into rivers, lakes or the air. Hence, the detection of these chemicals has become a cause of concern for researchers and scientists because they contribute largely to serious health problems. Herein, the potential of newly tailored nanomaterials for the detection of 2,4 dichlorophenoxyacetic acid (DCP) in humans was examined. The theoretical approach adopted in this work is within the framework of density functional theory (DFT) using the DFT/B3LYP-D3/def2SVP computational method. The reduction in the energy gap upon adsorption is indicative of good adsorbing properties. A chemisorption phenomenon was observed for DCP-GP/AlN. However, in most cases, physisorption occurs. Interestingly, the noncovalent nature of the interactions was observed in all the cases, indicating that the material was good. The green colour of the 3D RDG maps implies a significant intermolecular interaction. Sensor mechanisms confirmed that the nanocomposite materials exhibit excellent detection potential for DCP through greater charge transfer, better sensitivity, conductivity, and enhanced adsorption capacity. The potential of nanocomposite materials as stable and promising detectors for DCP pollutants was confirmed in this study. Hence, the studied GP/AlN nanocomposite material can be used in the engineering of future sensor devices for detecting DCP.

## Abstract 2

Despite their efficacy in eliminating undesired crops and increasing yield, a range of environmental issues and chronic ailments arise when hazardous chemicals are highly concentrated in wastewater and then deposited into rivers, lakes or the air.

## Graphical abstract

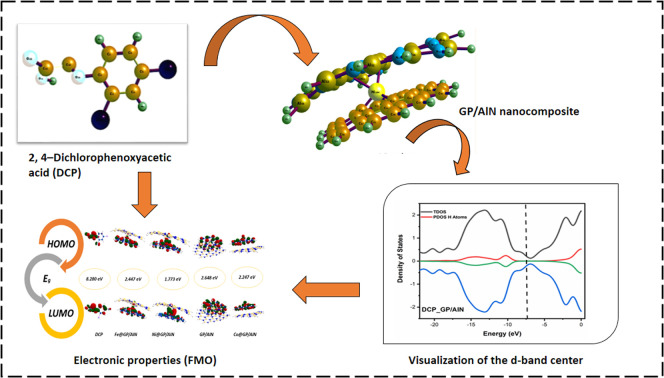

## 1 Introduction

Over the years, various anthropogenic activities have been recorded as nonpoint sources of pollution. In agriculture, the application of pesticides and herbicides poses a risk to human health, and major causes of soil, ground lake and water body contamination can be attributed to the intense consumption of these chemicals, such as 2,4-dichlorophenoxyacetic acid and glyphosate, in the management of poor yields.^1–4^ Despite the effectiveness of these chemicals in eliminating undesired crops and in turn increasing yield, a range of environmental issues and chronic ailments arise when these chemicals are highly concentrated in wastewater and then deposited into rivers, lakes or the air.^5,6^ 2,4-Dichlorophenol (DCP) is a derivative of phenol with a molecular formula of Cl_2_C_6_H_3_OH. It is crystalline in nature with a melting point of 450 °C and acts as an important precursor in the synthesis of herbicides, which include 2,4-dichlorophenoxyacetic acid (2,4-D) and other chlorinated chemicals.^7–9^ In research involving zebra fish, DCP tested positive for high toxicity due to its effect on the embryonic developmental process, causing morphological and behavioural defects in the offspring.^10^ DCP can enter the environment through the decomposition of 2,4-dichlorophenoxyacetic acid in contaminated soil, and DCP can reach water bodies during rainfall.^11,12^ During volatilization, DCP is introduced into the air and, when inhaled by humans, triggers nasal irritation and other cardiovascular problems.^13,14^ The effect of phenol-derived herbicides on weeds follows a mechanism of free radical formation, which induces the production of reactive oxygen species (ROS). These free radicals cause structural and cellular deformations of vital plant parts upon interaction with biomolecules such as carbohydrates and proteins.^15–17^ Phenoloxy chemicals are classified as highly toxic due to their ability to interrupt the cellular and enzymatic processes of various organisms and plants.^18,19^ Continuous exposure to DCP causes oxidative stress in hepatic tissues, contributing to an increase in kidney and renal problems.^20^ These observed and documented problems serve as catalysts for modern nanotechnology research, which targets various methods of modifying nanomaterials, such as graphene, for the effective sensing and adsorption of various pollutants, which poses a great risk to plants and humans.

Graphene has been one of the most commonly used carbon materials due to its unique chemical and physical characteristics, with increased hardness and strength compared to those of diamond and steel.^21,22^ It is made up of a number of carbon atoms arranged in a honeycomb-like shape and is usually single layered.^23^ One interesting attribute of graphene is its ability to be reinforced in various metal composites when subjected to modifications.^24–26^ Unlike some carbon nanotubes, graphene has a low density, high thermal conductivity and increased stiffness.^27–29^ Since *the* discovery of graphene by Novoselov *et al.* and the isolation of graphite through the tape stripping method, it has gained attention from various scientists and has been highly considered for application in several fields. Various issues in different fields have sparked the need to improve the features of materials for targeting these problems and developing sustainable solutions. A range of composite materials, such as aluminum–aluminum nitride, graphene–aluminum nitride and many others, have been discovered and are still being explored. Composite materials are composed of two or more constituents, one of which is a reinforcement material and the other of which is a matrix, often called a base material.^30,31^ The combination of these distinct materials aids in the modification of the behaviours and attributes to cause a dispersion of the material's use in different fields. Due to its outstanding properties of high thermal conductivity, large band gap, electrical sensitivity, shock resistivity, and mechanical strength.^32,33^ Due to these properties, aluminum nitride has been explored in this research. Over time, AlN has exhibited great potential and is used in the electrical and mechanical industries for the production of semiconductors, mainframes of supercomputers and field emission devices.^34–36^ It is believed that the potential of AlN has yet to be explored, increasing its potential for various testing and modifications. It has a melting point of 2500 °C or greater and can also exist in solid, liquid or gas states, allowing it to be produced through different nitridation methods.^37,38^

According to a literature review by Wang *et al.*, cetylpyridium chloride-modified pine sawdust (CPC-PS) exhibited better adsorption of bisphenol A and dichlorophenol (DCP) than PS (pine sawdust). Additionally, kinetic and thermodynamic analyses showed that BPA and DCP adsorption onto CPC-PS fit pseudo-second-order kinetics and the Freundlich model.^39^ In his work using an activated carbon, pine sawdust pyrolytic carbon (PSPC), to absorb 2,4-DCP and 4-CP, Song *et al.* reported that the process was spontaneous and that the adsorption capacity of PSAC was significantly reduced during the regeneration process of PSAC with ethanol. Their research introduced a viable method for the adsorption of environmental pollutants.^40^ Additionally, Lui *et al.* concluded that a graphene-based hyper crosslinked porous carbon composite (GN/HCPC) showed high adsorption for DCP and for other contaminants, such as tetracycline, phenol and bisphenol A, making the composite a practical and effective adsorbent for the removal of organic contaminants in wastewater.^41^ In this present research, a composite material was tailored following the combination of graphene and aluminum nitride. Graphene serves as the reinforcement material, and aluminum nitride, the base material, forms a composite material. For novelty, surface modification and exploration of the potential of these materials, the graphene material was doped with the transition metals Fe, Co, and Ni. A total of eight systems, consisting of four surfaces and four complexes, are understudied. Therefore, the objective of this study was to determine the system exhibiting the most suitable sensing potential towards DCP pollutant, in a bid to target the health problems arising from exposure to this pollutant from the inhalation or consumption of contaminated aquatic creatures and water.

## 2 Computational methodology

For the adsorption of 2,4-dichlorophenoxyacetic acid (DCP) on newly tailored nanocomposite materials, first-principles density functional theory (DFT) was used. The Gaussian 16 program^42^ and the GaussView 6.0.16 software package^43^ were used. The DFT/B3LYP-D3/def2SVP computational method was adopted in all calculations. Owing to the presence of heavy metals (Fe, Co, and Ni), the standard Los Alamos National Laboratory, including a double-zeta (LanL2DZ) basis set, was utilized.^44^ Prior to optimization, all structures were sketched and visualized on Gauss View 6.0.16 software to gain insight into the effects of adsorption on the morphology of the nanocomposite materials. Structural analysis, adsorption energy, quantum descriptor analysis, and topological studies were performed to examine the various adsorption behaviors of the newly tailored materials. The phenomenon of adsorption was observed from the adsorption energy calculated using eqn (1):

1 *E*_ad_ = *E*_Complex_ − (*E*_Surface_ + *E*_Molecule_)

where the energies of the adsorbate, surface and complex are denoted by *E*_Molecule_, *E*_Surface_, and *E*_Complex_, respectively. The required adsorption energy is denoted by *E*_ad_ in the equation. The *Multiwfn* 3.7 program^45^ was used to calculate the density of states (DOS) and the centre of the d-band, which were then plotted with Origin software.^46^ The Chemcraft 1.6 program^47^ was utilized to obtain the HOMO–LUMO plots. The 3D-RDG plot was visualized and generated using the VMD software package.^48^

## 3 Results and discussion

### 3.1 Geometry optimization

The geometry, such as bond lengths, were computed before adsorption to determine the effects of bond length on interactions. A molecule possessing shorter bond lengths is an indication of a strong force of attraction between the bonds, which reduces the reactivity of the systems understudied. On the other hand, weaker bond interactions are understood by longer bonds.^49,50^ Notably, many properties are determined by the bond length, such as the biological activity, polarity, phase of matter, and reactivity.^51^ Graphene has a monolayer (single layer) of carbon atoms tightly bonded in a hexagonal honeycomb, and AlN wurzite has a thermodynamically stable crystal structure. The optimized geometries of GP/AlN showed that graphene and wurzite face each other with carbon atoms connected to aluminium (Al) and (N). The bond length of the M–C bond strongly depends on the type of transition metal. Furthermore, there are significant difference in the M–C bond lengths in graphene nanocomposites.

**Fig. 1:**
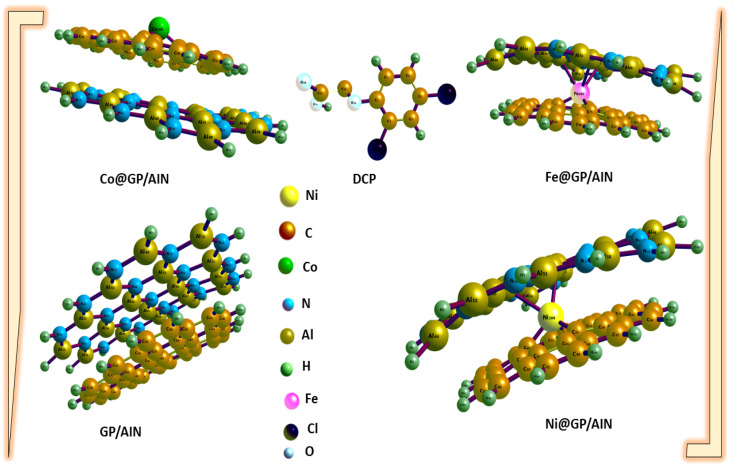
The optimized structures of the newly tailored nanocomposite materials and the 2,4-dichlorophenoxyacetic acid (DCP).

**Fig. 2:**
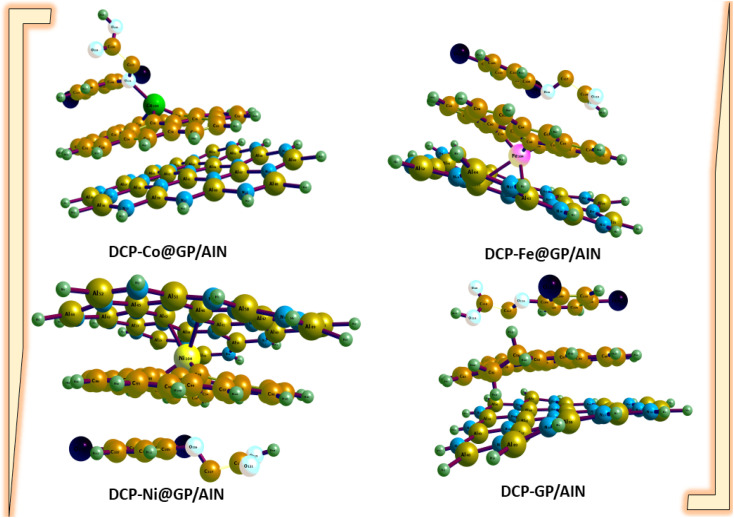
The optimized structures of the newly tailored nanocomposite materials upon the adsorption of 2,4-dichlorophenoxyacetic acid (DCP).

Table 1 shows the computed bond lengths between atoms of different elements (metal–C or Cl–C or N–C or O–C) before and after adsorption. Fig. 1 and 2 show the optimized structures of the studied surfaces and interactions. Fig. 1 shows the optimized structures of the studied surfaces and gases, namely, DCP, GP/AlN, Co@GP/AlN, Fe@GP/AlN, and Ni@GP/AlN. Fig. 2 shows the optimized structures of the studied interactions, namely, DCP-Co@GP/AlN, DCP-GP/AlN, DCP-Fe@GP/AlN, and DCP-Ni@GP/AlN.

**Table 1:** The computed bond lengths before and after adsorption were calculated *via* the DFT/B3LYP-D3/6-311G(d,p)/def2SVP computational method

Systems	Bond labels	Bond length	
		Before adsorption	After adsorption
DCP-GP/AIN	Al_41_–C_55_	4.089	4.088
	C_87_–Cl_114_		3.429
	N_25_–C_79_	3.469	3.488
	O_116_–C_79_		3.676
DCP-Co@GP/AIN	Al_42_–C_80_	3.535	3.529
	N_29_–C_65_	3.637	3.632
	Co_104_–O_116_		2.185
	Cl_115_–C_63_		3.952
DCP-Fe@GP/AIN	Fe_104_–N_25_	1.971	1.971
	Al_42_–C_60_	3.111	3.104
	Cl_114_–C_57_		3.402
	O_120_–C_55_		3.388
DCP-Ni@GP/AIN	Ni_104_–N_25_	2.042	2.043
	Al_42_–C_60_	3.002	3.382
	Cl_114_–C_56_		3.492
	*O*_*116*_ *–C*_*85*_		*3.245*

As shown in Table 1, in the DCP-GP/AlN interaction, there was a decrease in the Al_41_–C_55_ bond length after adsorption from 4.089 to 4.088 Å and an increase in the N_25_–C_79_ bond length from 3.469 to 3.488 Å. In DCP-Co@GP/AlN, there was a decrease in the Al_42_–C_80_ bond length from 3.535 to 3.529 Å and in the N_29_–C_65_ bond length from 3.637 to 3.632 Å. In DCP-Fe@GP/AlN, there was no change in the Fe_104_–N_25_ bond length of 1.971 Å, and there was a decrease in the Al_42_–C_60_ bond length from 3.111 to 3.104 Å. In DCP-Ni@GP/AlN, there is an increase in the Ni_104_–N_25_ bond length from 2.042 to 2.043 Å and in the Al_42_–C_60_ bond length from 3.002 to 3.38 Å.

### 3.2 Adsorption of 2,4-dichlorophenoxyacetic acid on the tailored nanocomposites

Due to the harmful effect of DCP on humans, the need to detect and adsorb it cannot be resolved. Herein, the molecule was adsorbed on a newly engineered surface tailored by first combining graphene and an aluminum nitride surface. Then, the graphene was modified by adding Fe, Co, and Ni dopants and further combined with the aluminum nitride. Altogether, four different adsorptions were obtained. The best adsorption configurations were ascertained during optimization, and the resulting complexes were confirmed to be in their best conformation. The results obtained from the computations are summarized in Table 2. Upon the adsorption of DCP on the GP/AlN nanocomposite, strong chemisorption occurred, which was attributed to the negative adsorption energy of −16.82 eV in the DCP-GP/AlN complex. However, adsorption on the nanocomposites formed by combining the AlN surface and structurally modified graphene results in weak and physical adsorption, which is best described as physisorption,^52^ as indicated by the accompanying positive adsorption energies of 15.55, 15.63, and 15.60 eV for DCP-Fe@GP/AlN, DCP-Co@GP/AlN, and DCP-Ni@GP/AlN, respectively (see Table 2). One can infer from the aforementioned results that the GP/AlN nanocomposite strongly adsorbs DCP, which makes it a suitable candidate for use as a sensor for DCP. On the other hand, the weak adsorption associated with the adsorption on the Fe@GP/AlN, Co@GP/AlN, and Ni@GP/AlN surfaces placed these surfaces as detectors for DCP molecules. Since the results obtained in this section are insufficient for determining the suitability of a material as a sensor or detector for 2,4-dichlorophenoxyacetic acid (DCP), further investigations have been carried out to investigate the electronic properties, nature of intermolecular interactions, effect of the d-band center and mechanisms of sensing.

**Table 2:** Summarized adsorption energies of the complexes calculated *via* the DFT/B3LYP-D3/6-311G(d,p)/def2SVP computational method

Complex	*E*_Complex_	*E*_Surface_	*E*_Gas_	*E*_Ads_ (Ha)	*E*_Ads_ (eV)
DCP-GP/AlN	−8285.659801	−6831.814691	−1453.226922	−0.61819	−16.82
DCP-Fe@GP/AlN	−9509.878038	−8057.222579	−1453.226922	0.571463	15.55
DCP-Co@GP/AlN	−9628.901013	−8176.248285	−1453.226922	0.574194	15.63
DCP-Ni@GP/AlN	−9754.448608	−8301.794786	−1453.226922	0.5731	15.60

#### 3.2.1 Toxicity of the materials

The potential toxicity for graphene due to their small size and ability to interact with biological systems. However, the specific toxicity depends on factors like surface, shape, size, functionalization, and exposure route. Aluminum nitride is generally considered to have low inherent toxicity. On the other hand, inhalation of AlN dust may lead to respiratory irritation. The metals dopants (Fe, Co, Ni) can be toxic in high concentrations. However, their specific toxicity depends on the metal, its oxidation state, and exposure route. Finally, the toxicity of the nanocomposite can be determined based on the aforementioned components stated in the following ways: size of particles and surface properties of the nanocomposite; the presence and type of metal dopant; and lastly the GP to AlN ratio.

### 3.3 Electronic properties

#### 3.3.1 HOMO–LUMO analysis

The information obtained from the HOMO−LUMO study pertains to the alteration in the electronic structural characteristics of the examined surfaces. This is mostly as a result of the disparities in energy between the HOMO and LUMO.^53^ The energy, strength, and stability of the surfaces and the complex following adsorption can be determined using the difference in energy between the two frontier molecular orbitals.^54^ Given that orbital hybridization corresponds to the bond formation of the GP/AlN nanocomposite and DCP gas molecule, the frontier molecular orbitals (FMOs) were analysed for pure GP/AlN and their equivalent complexes at their superior positions. Table 3 shows that DCP gas has the highest energy gap among all the studied systems. Among the studied surfaces, Ni@GP/AlN has the lowest energy gap value of 1.773 eV, which shows that it is the most reactive and least stable surface, followed by Co@GP/AlN at 2.247 eV, Fe@GP/AlN at 2.447 eV, and GP/AlN at 2.648 eV, which has the highest energy gap value, which shows that it is the least reactive and most stable surface. Among the studied interactions, DCP-Co@GP/AlN has the lowest energy gap value of 1.979 eV, followed by DCP-GP/AlN at 2.282 eV, DCP-Fe@GP/AlN at 2.335 eV, and DCP-Ni@GP/AlN at the highest energy gap value of 10.073 eV, which shows that it is the most stable and least reactive interaction.^55^ This result shows that upon adsorption, the rate of reactivity decreased. Table 3 shows the ionization potential (IP), electron affinity (EA), chemical softness (*S*), chemical hardness (*η*), electronegativity (*X*), chemical potential (*μ*), and electrophilicity index (*ω*) values of the nine studied systems. These descriptors were computed based on Koopmans's approximation and are presented in eqn (2)–(8).

2 Ionization energy (I.E.) = −*E*_HOMO_

3 Electron affinity (E.A.) = −*E*_LUMO_

4
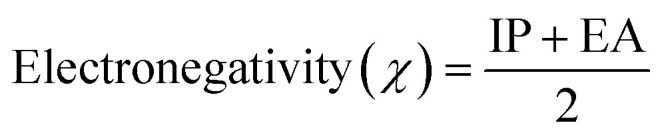
5
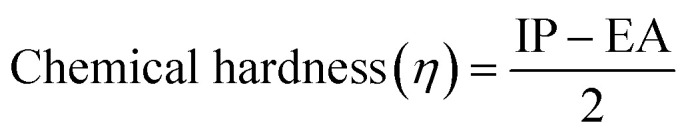
6
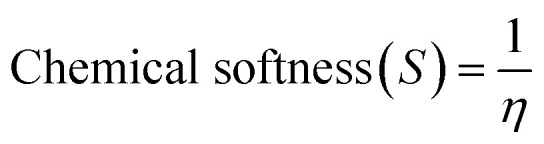
7
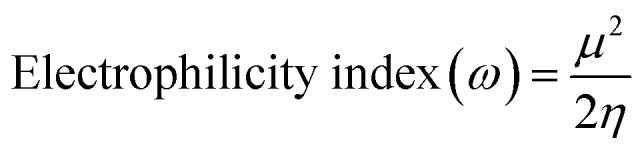

8 Electrochemical potential (*μ*) = −*χ*

**Table 3:** The quantum descriptors were calculated *via* the DFT/B3LYP-D3/6-311G(d,p)/def2SVP computational method. All units are in electron volts (eV), except for chemical softness (*S*), which has a unit of eV^−1^

Systems	*E*_HOMO_	*E*_LUMO_	*E*_g_	IP	EA	*μ*	*η*	*S*	*ω*	*E*_FL_
DCP	−9.145	−0.865	8.280	9.145	0.865	5.005	4.140	0.241	3.025	−5.005
GP/AlN	−4.887	−2.239	2.648	4.887	2.239	3.563	1.324	0.755	4.793	−3.563
Fe@GP/AlN	−4.900	−2.453	2.447	4.900	2.453	3.677	1.224	0.817	5.524	−3.677
Co@GP/AlN	−5.469	−3.222	2.247	5.469	3.222	4.345	1.124	0.889	8.402	−4.345
Ni@GP/AlN	−4.747	−2.974	1.773	4.747	2.974	3.861	0.886	1.129	8.406	−3.861
DCP-GP/AlN	−4.828	−2.546	2.282	4.828	2.546	3.687	1.141	0.876	5.957	−3.687
DCP-Fe@GP/AlN	−6.253	−3.917	2.335	6.253	3.917	5.085	1.168	0.856	11.072	−5.085
DCP-Co@GP/AlN	−5.216	−3.236	1.979	5.216	3.236	4.226	0.989	1.011	9.019	−4.226
DCP-Ni@GP/AlN	−5.883	−4.190	1.693	5.883	−4.190	0.846	5.036	0.198	0.071	−0.846

**Fig. 3:**
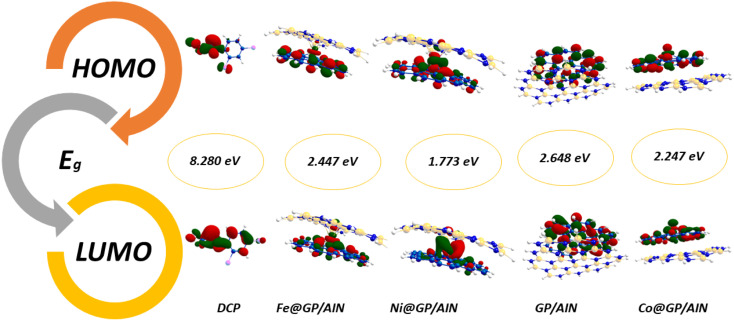
HOMO and LUMO plots for the nanocomposite materials prior to adsorption.

**Fig. 4:**
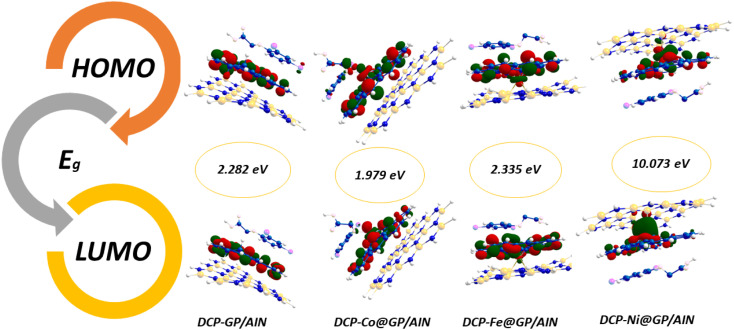
HOMO and LUMO plots for the nanocomposite materials upon adsorption.

The ionization potential of the studied surfaces falls within a close range of 4.747–5.469 eV. Also, Co@GP/AlN has the highest ionization potential value of 5.469 eV, whereas the lowest value of 4.747 eV was observed for Ni@GP/AlN. Then, followed by GP/AlN with the value of 4.887 eV, and Fe@GP/AlN with a value of 4.900 eV. Moreover, electron affinity ranges from 2.239 to 3.222 eV, with Co@GP/AlN associated with the highest peak of 3.222 eV, then followed by Ni@GP/AlN with a value of 2.974 eV, and values of 2.453 and 2.239 eV for GP/AlN and Fe@GP/AlN respectively. Fig. 3 and 4 show the HOMO and LUMO plots prior to and upon adsorption, respectively. The plots illustrate the localization of the HOMO and LUMO at sites on the adsorbate and adsorbent.

#### 3.3.2 NBO analysis

NBO analysis is among the quantum chemical techniques for examining chemical bonds, and the degree of charge transfer between Lewis-type donor orbitals and non-Lewis acceptor orbitals in molecular systems.^56^ A large value of *E*^2^ reflects a stronger inclination from an electron donor to an electron receptor, as a result, a greater degree of conjugation of the entire system.^57^ They also show a significant connection between the donor orbitals (*i*) and the receptor orbitals (*j*). As presented in Table 4, Ni@GP/AlN has the highest stabilization energy of 959.5 kcal mol^−1^ among the studied surfaces, which shows that it is the most stable surface and can be easily stabilized. This is attributed to the charge transfer resulting from charge delocalization from the antibonding character of the lone pair LP*(1) C_70_ to the same antibonding character of the lone pair LP*(1) C_71_. The GP/AlN nanocomposite has the least stabilization energy of 107.6 kcal mol^−1^, showing that it is the least stable surface among its counterparts. Among the studied interactions, DCP-GP/AlN has the highest stabilization energy of 1284.41 kcal mol^−1^, which shows that it is the most stable surface upon adsorption, and it is attributed to the charge transfer resulting from charge delocalization from the antibonding character of the lone pair LP*(1) C_69_ to the same antibonding character of the lone pair LP*(1) C_59_. Notably, the least stabilization energy was observed in DCP-Ni@GP/AlN upon adsorption. The trend of stabilization energy of the studied interactions follows: DCP-GP/AlN > DCP-Fe@GP/AlN > DCP-Co@GP/AlN > DCP-Ni@GP/AlN.

**Table 4:** NBO analysis results calculated *via* the DFT/B3LYP-D3/6-311G(d,p)/def2SVP computational method

System	Donor	Acceptor	*E* ^2^ (kcal mol^−1^)	*E*(*j*) − *E*(*i*) (a.u.)	*F*(*i*,*j*) (a.u.)
DCP	π* (C_2_–C_3_)	π* (C_4_–C_5_)	394.79	0.01	0.099
GP/AlN	π* (C_82_–C_83_)	σ* (N_22_–C_92_)	107.60	0.01	0.065
Co@GP/AlN	LP (4) Co_104_	LP (1) C_56_	692.58	0.06	0.217
Fe@GP/AlN	LP (1) C_72_	LP* (1) C_69_	910.65	0.01	0.131
Ni@GP/AlN	LP* (1)C_70_	LP* (1) C_71_	959.50	0.01	0.148
DCP-GP/AlN	LP (1) C_69_	LP* (1)C_59_	1284.41	0.01	0.146
DCP-Co@GP/AlN	LP* (1) C_97_	LP (1) C_94_	204.69	0.02	0.084
DCP-Fe@GP/AlN	LP (1) C_85_	LP* (1) C_79_	1033.17	0.02	0.161
DCP-Ni@GP/AlN	π* (C_109_–C_110_)	π* (C_105_–C_106_)	139.95	0.01	0.080

### 3.4 Effect of the d-band center

**Fig. 5:**
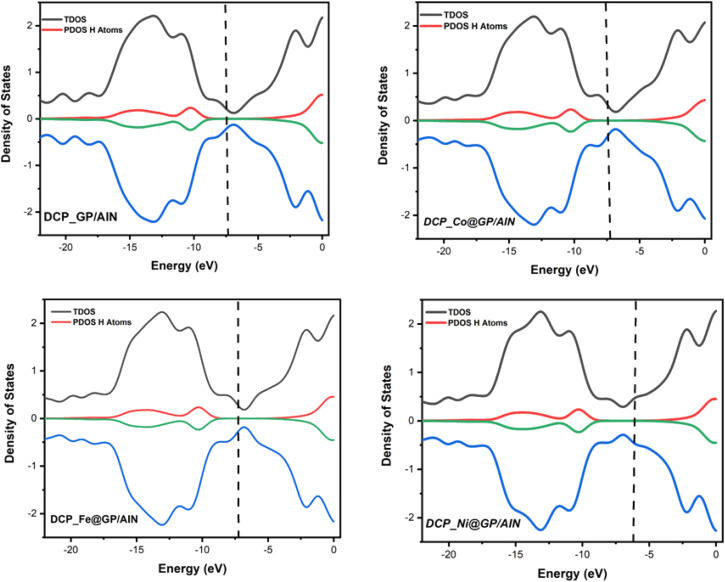
Visualization of the d-band center plots for the studied systems.

The d-band center is an important analysis used in gaining knowledge regarding the variation in small-molecule chemisorption strength on transitional metals.^58^ The vertical line in this instance is a common visual showing the highest occupied molecular orbital (HOMO) level. The lowest level of unoccupied molecular orbitals (LUMOs) is correlated with the center of the total density of states (TDOS). The difference between the center of the TDOS and the vertical line corresponding to the HOMO is the d-band center.^59,60^ As shown in Table 5, DCP-Co@GP/AlN has the highest d-band center value of −2.228 eV, which shows that there is a high rate of adsorption of DCP gas on the Co@GP/AlN surface, while DCP-Ni@GP/AlN possess the lowest d-band center value of −3.637 eV, which shows that there is a low rate of adsorption of the studied gas on the surface. It can be observed that, the d-band center result is in agreement with the adsorption rate, thereby reflecting the existence of the interaction between the surface and gas. Fig. 5 presents the visualization of the systems under the context of the d-band center effect.

**Table 5:** The d-band center values of the studied systems calculated *via* the DFT/B3LYP-D3/6-311G(d,p)/def2SVP computational method

System	Center of TDOS (eV)	Center of PDOS (eV)	Vertical dash line (eV)	d-Band center (eV)
DCP-GP/AlN	−10.102	−8.674	−7.637	−2.465
DCP-Co@GP/AlN	−9.936	−8.766	−7.708	−2.228
DCP-Fe@GP/AlN	−9.948	−8.654	−7.564	−2.384
DCP-Ni@GP/AlN	−9.941	−8.562	−6.304	−3.637

### 3.5 Visual study

#### 3.5.1 QTAIM analysis

As posited by Bader *et al.*, the quantum theory of atoms-in-molecules investigates the critical position of bonds to explore the nature of interactions in the systems under study.^61^ The QTAIM encompasses the intricacies of interaction nature and, similarly, explains other parameters for an accurate and in-depth study. The structural properties alone may not sufficiently depict the behavioral aspects of the molecules and the distinct features of the interactions. These variations in the bond at different critical bond points contribute to the uniqueness observed in structural and electronic properties, *etc.*^62^ Additionally, the charge density extrema can be evaluated through QTAIM analysis. Multiwfn software was utilized to effectively visualize these bonds and determine various parameters in this topological analysis. The calculated electron density, Lagrangian kinetic energy *G*(*r*), energy density, *H*(*r*), *G*(*r*)/*V*(*r*), *etc.*, are summarized in Table 6. The nature of interactions can be categorized into covalent, noncovalent and partial covalent bonds and can be observed by the values of the Laplacian of electron density ∇^2^*ρ*(*r*) and *H*(*r*). According to previous literature, partial covalent, covalent and noncovalent interactions occur when ∇^2^*ρ*(*r*) > 0 and *H*(*r*) < 0, ∇^2^*ρ*(*r*) < 0 and *H*(*r*) > 0 and ∇^2^*ρ*(*r*) > 0 and *H*(*r*) > 0, respectively.^63,64^

**Table 6:** The topological parameters calculated *via* the DFT/B3LYP-D3/6-311G(d,p)/def2SVP computational method^a^

System	Bonds	CPs	*P*(*r*)	∇^2^*ρ*(*r*)	*G*(*r*)	*V*(*r*)	*H*(*r*)	ELF	LOL	*G*(*r*)/*V*(*r*)	*λ*_1_	*λ*_2_	*λ*_3_	*λ*_1_/*λ*_3_	*E*
DCP@GP/AlN	C_82_–N_28_	177	0.369	0.111	0.203	−0.130	0.739	0.150	0.110	−1.562	−0.197	−0.519	0.136	−1.449	2.797
	C_72_–Al_36_	341	0.628	0.149	0.306	−0.240	0.656	0.385	0.167	−1.275	−0.230	−0.882	0.180	−1.278	1.607
	Cl_115_–H_123_	131	0.477	0.171	0.326	−0.226	0.101	0.138	0.106	−1.442	−0.271	−0.374	0.236	−1.148	0.379
DCP-Fe@GP/AlN	H_76_–N_28_	158	0.417	0.135	0.250	−0.162	0.882	0.150	0.110	−1.543	−0.953	−0.182	0.163	−5.847	0.911
	O_116_–C_60_	210	0.531	0.222	0.409	−0.262	0.147	0.127	0.102	−1.561	−0.182	−0.371	0.278	−0.655	1.036
	Cl_115_–C _105_	192	0.571	0.178	0.369	−0.293	0.764	0.196	0.124	−1.259	−0.243	−0.324	0.235	−1.034	0.335
DCP-Co@GP/AlN	N_118_–H_102_	218	0.336	0.116	0.204	−0.117	0.872	0.110	0.960	−1.743	0.141	−0.178	−0.703	−0.201	1.526
	N_119_–C_95_	247	0.453	0.154	0.289	−0.193	0.961	0.149	0.110	−1.497	−0.134	−0.208	0.188	−0.713	0.561
	Co_104_–Cl_114_	280	0.154	0.227	0.819	−0.107	−0.252	0.993	0.249	−7.654	−0.928	0.376	−0.563	1.648	0.648
DCP-Ni@GP/AlN	C_81_–Al_44_	211	0.625	0.141	0.307	−0.262	0.453	0.374	0.165	−1.172	−0.176	−0.233	0.182	−0.967	0.325
	N_22_–N_18_	139	0.497	0.151	0.290	−0.201	0.884	0.199	0.125	−1.443	−0.149	−0.274	0.194	−0.768	0.842
	Cl_115_–H_113_	248	0.646	0.194	0.411	−0.336	0.749	0.238	0.135	−1.223	−0.284	−0.390	0.262	−1.084	0.374

a The units of the parameters in Table 5 are as follows: *P*(*r*), *e*/Ang^3^; ∇^2^*ρ*(*r*), *e*/Ang^3^; *λ*_1_, *λ*_2_, and *λ*_3_ have a unit of *e*/Ang^5^ respectively; *G*(*r*) and *V*(*r*) have units of eV; *H*(*r*) has a unit of eV Ang^3^/*e*; ellipticity of electron density (*ε*), electron localization function (ELF), *G*(*r*)/*V*(*r*) and *λ*_1_/*λ*_3_ are dimensionless.

In this study, all the complexes exhibited noncovalent interactions except for the Co_104_–Cl_114_ bond in the DCP-Co@GP/AlN complex, which is partially covalent. The *P*(*r*) and ∇^2^*ρ*(*r*) values confirm the existence of a noncovalent nature of the interaction since all the values are greater than zero (>0). Consequently, the *λ*_1_/*λ*_3_ values for the complexes DCP@GP/AlN, DCP-Fe@GP/AlN, DCP-Co@GP/AlN and DCP-Ni@GP/AlN, as reported in Table 6, are all less than that of the H_76_–N_28_ bond in DCP-Fe@GP/AlN, which has a smaller negative value of −5.847 a.u. and indicates the strong presence of intermolecular interactions across all the complexes. Furthermore, a great degree of stability in the interaction and charge distribution is confirmed by the domination of the small values of bond ellipticity. Additionally, a previous literature review established that when the *G*(*r*)/*V*(*r*) values are greater than one, a covalent interaction occurs, and partial covalent and noncovalent interactions occur when the values are between 1 and 0.5 and less than 0.5, respectively.^65,66^ The *G*(*r*)/*V*(*r*) further confirms the presence of noncovalent interactions since all values are less than one. According to the analysed parameters, all the complexes exhibit a degree of stability and a noncovalent nature of interaction, which is required for outstanding sensor performance.

#### 3.5.2 3D RDG plots

**Fig. 6:**
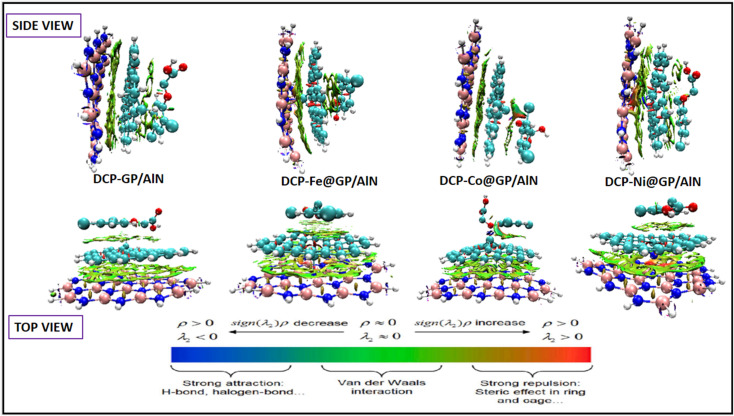
3D-RDG plots of the complexes in two views, namely, the side view and top view.

Understanding the concept surrounding the 3D isosurface of systems is made possible by studying noncovalent interactions, which is often referred to as reduced density gradient (RDG) analysis. This analysis explores the interaction within and between the adsorbate and the adsorbent in a study. Its application spans various fields, such as medicinal chemistry and computational studies.^67^ These interactions do not promote the transfer of electrons but follow a colouration pattern. In previous studies, various forces are represented in a colour range, which are shown as scattered patches or lobes in some cases around the isosurface.^68^ NCI analysis focuses on the study of weak interactions, the van der Waals force of attraction, which is confirmed when green-colored lobes or patches are found around the interaction area of a complex. The blue-colored region depicts the strong presence of hydrogen bonding, and the red areas indicate the existence of steric interactions. These interactions affect the behaviors of the individual complexes, causing variation across them upon interaction.^69,70^Fig. 6 shows all the computationally visualized isosurfaces of the studied complexes. As represented in the figure, all complexes are observed to have a large green isosurface scattered around the composite material area and the pollutant area, implying significant intermolecular interactions. The DCP@GP/AlN complex exhibited only van der Waals forces of attraction, and the DCP-Fe@GP/AlN, DCP-Co@GP/AlN and DCP-Ni@GP/AlN complexes were dominated by green iso-surfaces and a slight red colour around some regions, indicating the presence of strong van der Waals forces between the molecules of the adsorbate and adsorbent and slight steric forces within the composite material. The observed trends in the isosurface indicate that the DCP@GP/AlN complex is most suitable and possesses a very weak interaction, although other complexes show great potential in the sensing and adsorption of the 2,4-dichlorophenoxyacetic acid (DCP) pollutant, which is a precursor in the formation of herbicides and pesticides.

### 3.6 Sensing mechanisms

#### 3.6.1 Work function and electrical conductivity

According to the Kevin probe,^71^ when the work function of a semiconductor changes, it affects the electron density of that material; as a result of the instability of the emission properties, an electrical signal is produced. The greatest changes in the work functions of −0.7808 and 0.3831 were observed for the Ni@GP/AlN and Fe@GP/AlN materials, respectively, indicating greater changes in the electron density of the materials and greater electrical signals, as provided by the Kevin probe. The high work functions of 5.09, 4.23, and 3.69 eV are attributed to DCP-Fe@GP/AlN, DCP-Co@GP/AlN, and DCP-GP/AlN, respectively (see Table 7). It is noteworthy to mention that greater work function indicates high performance of a material comparatively. Hence, this attribute is an indicator of a material suitable for designing detector and sensor devices.

**Table 7:** Sensor mechanisms calculated by DFT/B3LYP-D3/6-311G(d,p)/def2SVP

Systems	*ϕ* (eV)	Δ*ϕ* (eV)	*Q*_*t*_ (e)	Δ*N*	Δ*E*-back-donation (eV)	% Δ*E*_g_
DCP-GP/AlN	3.69	0.0349	0.220	0.011387	−0.28528	−13.83
DCP-Fe@GP/AlN	5.09	0.3831	1.149	0.039285	−0.29194	−4.56
DCP-Co@GP/AlN	4.23	−0.0276	0.995	−0.00802	−0.24749	−11.90
DCP-Ni@GP/AlN	0.85	−0.7808	1.125	6.254571	−1.25911	−4.52

The conduction of electricity is an attribute of a good sensor material. The energy gap can be related to the electrical conductivity of a material *via*eqn (9) in such a way that a higher energy gap results in a higher conductivity and *vice versa*.^72–74^ Theoretically, electrical conductivity is calculated using eqn (9), where the constant, temperature, energy gap, and Boltzmann constant are denoted by *K*, *T*, *E*_g_, and *B*, respectively.

9 *σ* = *AT*^2/3^e^(*E*_g_/2*KT*)^

The percentage changes in the energy gap, % Δ*E*_g_, computed are summarized in Table 7, wherein a decrease in the energy gap was observed upon adsorption (indicator of increased reactivity). The greatest percentage change in the *E*_g_ of DCP-GP/AlN and DCP-Co@GP/AlN were −13.83 and −11.90%, respectively. Similarly, DCP-Fe@GP/AlN and DCP-Ni@GP/AlN showed similar percentage changes of −4.56 and −4.52, respectively (see Table 7). A greater negative percentage is an indication of increased reactivity of the system. As provided in Subsection 3.3.1 of the quantum descriptors, the increasing order of the energy gap is an increasing pattern of conductivity, which follows the order DCP-Ni@GP/AlN < DCP-Co@GP/AlN < DCP-Fe@GP/AlN < DCP-GP/AlN. This can be understood so that the least and greatest conductivity are found in the adsorption of DCP on Ni@GP/AlN and GP/AlN surfaces respectively. In furtherance, the GP/AlN surface without doping modification happened to have the least reactivity and most conducting. Lastly, since the energy gap falls within a close range of 1.693 to 2.335 eV, the electrical conductivities among the investigated surfaces are relatively close.^75^

#### 3.6.2 Backdonation and FET patterns

Based on Pearson theory,^76^ electrical back-donation can be defined using a mathematical formula in such a way that chemical hardness is used therein. The eqn (10) shows the expression of electrical back donation in terms of chemical hardness.10
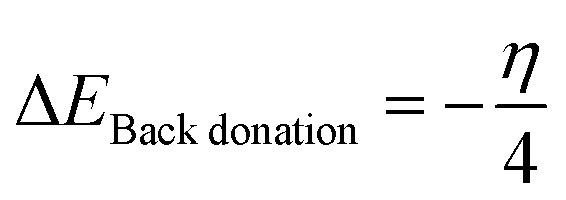


Based on literature reviewed, negative values of Δ*E*_Back donation_ are indicative of good material. That is, those showcasing higher negative magnitude possesses an inherent adsorbing property that grants such material the gateway to be used in coupling a sensor device.^77^ As observed from the table, all studied systems are attributed to negative back-donation, with the highest negative value of −1.25911 eV for DCP-Ni@GP/AlN, indicating good material performance. Other studied systems are within a close range of −0.29194 to −0.24749 eV, depicting a relatively good sensing property. The electron mobility from the nanocomposite material to the DCP molecule can be elucidated using the fraction of electron transfer (FET).^78^ The Peason theory is used to calculate the electronegativity (*χ*) and chemical hardness (*η*) of FETs. The FET can be computed *via*eqn (11).11
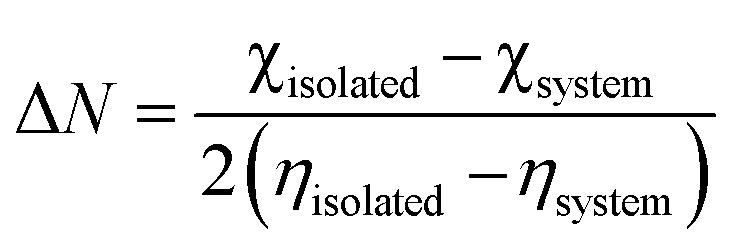


Thorough literature survey shows that lower *η* value is attributed to greater Δ*N* value, which in turn causes an increase in FET upon adsorption.^79^ A high FET indicates the movement of electrons from the nanocomposite materials to the DCP molecule.^80^ The highest FET value of 6.254571 is attributed to DCP-Ni@GP/AlN. Additionally, relatively high FETs of 0.011387 and 0.039285 corresponding to DCP-GP/AlN and DCP-Fe@GP/AlN, showcased good adsorption behaviour.

#### 3.6.3 Effect of charge transfer, *Q*_NBO_

The use of natural charge on an adsorbent material and adsorbate is an important concept in determining the charge transfer within a system. Generally, a positive magnitude of *Q*_NBO_ indicates charge transfer from the nanocomposite to the DCP molecule, whereas a transfer from the DCP molecule to the nanocomposite can be elucidated from the negative *Q*_NBO_.^81,82^ Using the mathematical relation in eqn (12), the charge transfer can be calculated.

12 *Q*_NBO_ = *Q*_adsorption_ − *Q*_isolated_

Owing to the positive charge transfer observed in all cases, electrons are transferred from the nanocomposite to the DCP molecule. This scenario elucidates the presence of an excellent adsorbing materials.^83^

### 3.7 Computational insights and future directions

The theoretical investigation carried out in this study will help in the future experiments through screening new materials, optimizing adsorbent design, and tailoring the surface properties. Due to large library of potential materials, DFT model can be employed virtually to screen this large library. In the long run, the DFT investigation will guide in selecting the most promising candidates for further experimental study, while saving time and resources. Additionally, the optimization of the structure and composition of the adsorbent at the atomic level can be achieved by using the newly modelled materials. In line with this, one can simulate different configurations of metal dopants within the GP/AlN nanocomposite framework in order to identify the most effective arrangement for the adsorption of DCP. Lastly, this theoretical investigation creates room for the ease and flexibility of tailoring surface properties by analyzing the interaction between the DCP and the model adsorbent. With this, one can gain useful insight into the underlying mechanisms of adsorption. Conclusively, the knowledge gained in this computational inquiry can be a guide when tailoring the surface properties of the adsorbent for even stronger and highly selective detection of DCP.

The feasibility in the synthesis of these materials can be accounted for by firstly putting into consideration, how the knowledge gained can be of use in the experimental. One among many others is the identification of active sites, where specific sites on the adsorbent materials with the most favourable for the adsorption of DCP can be pinpointed by the model. This aspect helps in experimental characterization practices on the active sites, thereby creating a deeper understanding of the adsorption process. Experimental observations like adsorption energy can be interpreted using this theoretical inquiry to better understand adsorption mechanism at the molecular level. Lastly, from the loaded adsorbent, desorption process of the adsorbate can be aided by this model, providing insights into the regeneration conditions usually required for efficient reusability of the adsorbent.

It is important to note that, as this model is a simplification of reality, the need for experimental validation cannot be jettisoned. This knowledge can be used to design experiments with techniques like scanning electron microscopy (SEM) or X-ray photoelectron spectroscopy (XPS) to focus on these active sites and validate the model's predictions. This information can also guide further experiments like temperature-programmed desorption (TPD) to measure the energy required to desorb DCP and validate the predicted mechanism, and many among others.

## 4 Conclusions

The intricacies of DCP molecule adsorption on newly tailored nanocomposite materials were explored in the present study. Using density functional theory, this study provides new insight into the adsorption potential of GP/AlN, Fe@GP/AlN, Co@GP/AlN, and Ni@GP/AlN nanocomposite surfaces for the detection of 2,4-dichlorophenoxyacetic acid (DCP) in humans. Knowledge on the chemistry of adsorption has been gained using various theoretical analyses, such as structural analysis, FMO and NBO analyses, determination of the effect of the d-band center, QTAIM and 3D RDG analyses, adsorption studies, and sensor mechanism studies, among others. The following conclusions are drawn:

I. As adsorption occurred, changes in the morphology of the nanocomposite materials were observed, as indicated by the contraction and stretching of bond lengths.

II. The phenomena of adsorption observed in most cases are physisorption and chemisorption for DCP-GP/AlN. This result demonstrated the suitability of the nanocomposite materials as detectors for DCM.

III. In all cases, the energy gap decreased upon adsorption, which is indicative of good sensor materials. During post-adsorption, the energy gap values were computed to be within a relatively close range of 1.693 to 2.336 eV.

IV. According to the QTAIM analysis, all the complexes exhibit a certain degree of stability and noncovalent interactions, which are required for outstanding sensor performance.

V. The green colour of the 3D RDG maps implies a significant intermolecular interaction. The DCP@GP/AlN complex exhibits only van der Waals forces of attraction, and the DCP-Fe@GP/AlN, DCP-Co@GP/AlN and DCP-Ni@GP/AlN complexes are dominated by green iso-surfaces, and a slight red colour appears around some regions, indicating the presence of strong van der Waals forces.

VI. Sensor mechanisms affirmed that the nanocomposite materials exhibit excellent detection potential for DCP through greater charge transfer, better sensitivity, conductivity, and enhanced adsorption capacity.

## Data availability

All data are contained within the manuscript.

## Author contributions

Chioma G. Apebende: project conceptualization, design and supervision. Ismail O. Amodu: methodology, validation, investigation, analysis. Miracle N. Ogbogu: writing, editing and data curation; Monsurat Alarape Raimi: review, editing and manuscript final draft; and Godwin O. Igomah: validation, methodology, reading, and editing.

## Conflicts of interest

The authors declare no financial or competing interests.
